# Homologous Recombination Occurs in *Entamoeba* and Is Enhanced during Growth Stress and Stage Conversion

**DOI:** 10.1371/journal.pone.0074465

**Published:** 2013-09-30

**Authors:** Nishant Singh, Alok Bhattacharya, Sudha Bhattacharya

**Affiliations:** 1 School of Environmental Sciences, Jawaharlal Nehru University, New Delhi, India; 2 School of Life Sciences, Jawaharlal Nehru University, New Delhi, India; George Washington University School of Medicine and Health Sciences, United States of America

## Abstract

Homologous recombination (HR) has not been demonstrated in the parasitic protists *Entamoeba histolytica* or *Entamoeba invadens*, as no convenient method is available to measure it. However, HR must exist to ensure genome integrity, and possible genetic exchange, especially during stage conversion from trophozoite to cyst. Here we show the up regulation of mitotic and meiotic HR genes in *Entamoeba* during serum starvation, and encystation. To directly demonstrate HR we use a simple PCR-based method involving inverted repeats, which gives a reliable read out, as the recombination junctions can be determined by sequencing the amplicons. Using this read out, we demonstrate enhanced HR under growth stress in *E. histolytica*, and during encystation in *E. invadens*. We also demonstrate recombination between chromosomal inverted repeats. This is the first experimental demonstration of HR in *Entamoeba* and will help future investigations into this process, and to explore the possibility of meiosis in *Entamoeba*.

## Introduction

*Entamoeba histolytica*, the parasitic protist which causes amoebiasis in humans, is distributed worldwide, with greater prevalence in developing countries. It is estimated to result in ∼70,000 deaths annually [1], [2]. It has a simple life cycle consisting of the dormant, tetranucleate cyst which, under favourable conditions, converts into the actively dividing, uninucleate trophozoite. The latter undergoes encystation under adverse growth conditions. These differentiation stages can be reproduced in lab conditions in the reptilian parasite *Entamoeba invadens*, but encystation has not yet been achieved in axenically grown *E. histolytica* trophozoites. *E. invadens* is therefore used as a model system for differentiation studies in *Entamoeba* [3], [4]. Several studies have been conducted on the cell cycle and changes in number of nuclei and DNA content during encystation [5]–[8]; however DNA exchange by recombination has not been reported in *Entamoeba*.

The genome of all organisms is under constant attack from endogenous metabolic processes and exogenous environmental factors that can alter its chemical structure. DNA damage can lead to mutations, deletions, insertions, translocations, and loss of essential genetic information. It is therefore of vital importance that cells should repair these lesions accurately [9], [10]. Parasitic protists are continuously exposed to environmental toxins and host immune pressure, which can affect the stability of their genome. However, DNA repair mechanisms have not been investigated in detail in most of these organisms.

In eukaryotic and prokaryotic cells, homologous recombination (HR) is an important repair mechanism to eliminate DNA double strand breaks (DSBs) and to bypass lesions that block the replicative polymerase during DNA synthesis [11]. In addition it is an important mechanism to generate genetic diversity used by parasites to evade the host immune response [12], [13]. Central to HR is homologous pairing and DNA strand exchange. Most of the investigations related to meiotic and mitotic genes have been carried out in model organisms ranging from animals, fungi and plants. Based on the knowledge from *Saccharomyces cerevisiae* a brief description of the conserved events is as follows. HR is initiated by DNA DSBs which could be caused due to DNA damage or during meiosis by the *SPO11* endonuclease [14], [15]. DSBs are nucleolytically processed by the Mrn/Mrx complex, which includes Mre11p, to generate single strand tails with 3′-OH ends. The ssDNA associates with specific proteins (Rad51p and Dmc1p) to form a nucleoprotein filament [16] which is stimulated by the Hop2-Mnd1 proteins to invade a homologous sequence and form a D-loop intermediate [17]. The Msh2 protein (which interacts with Mlh1p) is part of a complex involved in strand invasion. Subsequent steps lead to the capture of the second DSB end and the formation and resolution of Holliday junctions, promoted by the Msh4, Msh5 and Mer3 proteins [18]. A comprehensive list of genes involved in meiotic and mitotic recombination has been compiled from a variety of eukaryotic organisms [19], , and has been used to search for homologs in protists like *Giardia lamblia* which is not known to have a sexual cycle. Unlike in model systems like yeast and human it may not yet be possible to characterize these genes by genetic and biochemical analysis in most of the parasitic protists. However, their identification in these organisms has now become possible, and is greatly facilitated by the availability of genome sequence data. The presence of different meiotic and HR specific genes in genome sequences suggests the possibility of DSBs and inter-homolog recombination and Holliday junction resolution with crossover interference in many protists [19], [20]. In *E. histolytica*, some meiotic genes like *SPO11, DMC1, MND1*, and many HR specific genes like *MLH1, MSH2, RAD21*, and *RAD51* are present [19], [20]. The *E. histolytica* genome also contains *RAD52* epistatic group related genes, which differentially express when DSBs are induced by UV-C irradiation [21].

Stage inter-conversion between the trophozoite and cyst is essential to disease transmission and pathogenesis in *E. histolytica* [22]. This could also be a stage where the organism may engage in genetic recombination. However, this has not been investigated in *Entamoeba*. In the protozoan parasite *Giardia intestinalis*, exchange of genetic material and expression of many HR and meiosis-specific genes has been observed during encystation. The expression of GFP-tagged *DMC1, SPO11* and *HOP1* was observed in the cyst nuclei of *Giardia* but not in the nuclei of trophozoites. Nuclear fusion and exchange of genetic material was elegantly demonstrated in *Giardia* during the course of cyst formation [23], [24].

HR is also reported in other protozoan parasites like *Trypanosoma*, *Plasmodium*, and *Leishmania* [12], [13], [25]. *P. falciparum* has homologs for *RAD51, MRE11, RAD54* which are probably involved in the extensive HR-mediated DNA rearrangements exhibited by this parasite [26]. *Trypanosoma* and *Plasmodium* use HR to protect themselves from host immune response by inducing antigenic variation [12], [13].

Ploidy changes and unscheduled gene amplification have been reported in *Entamoeba* [27]–[30]. These processes may be linked to genetic recombination. Here we present the first direct demonstration of HR in *Entamoeba* using a construct with inverted repeats, which upon recombination results in sequence inversion. Using this read out, we found that HR is enhanced under stress conditions in *E. histolytica*, and during encystation in *E. invadens*.

## Methods

### Strains and cell culture

All experiments were carried out with *E. histolytica* strain HM-1:IMSS. The cells were maintained and grown in TYI-S-33 medium supplemented with 15% adult bovine serum, Diamond's vitamin mix and antibiotics (0.3 units/ml penicillin and 0.25 mg/ml streptomycin) at 35.5°C. To achieve serum starvation, medium from mid log phase grown *Entamoeba* cells was replaced with TYI-33 medium containing 0.5% adult bovine serum for indicated time periods. Heat stress was given for 20, 40 and 60 min at 42°C. For oxygen stress cells were grown for 20, 40 and 60 min in 10 ml TYI-S-33 medium in 50-ml flask. G-418 (Sigma) was added at 10 mg/ml for maintaining the transfected cell lines.

*E. invadens* strain IP-1 was obtained from the American Type Culture Collection and maintained at 25°C in TYI-S-33 containing 15% heat inactivated adult bovine serum,125 µl/100 ml streptomycin/penicillin G and 2.0% vitamin mix [31].

### Cyst induction and excystation

This was done essentially as described [4] and adapted in our lab [8]. Briefly, log phase trophozoites grown in 50 ml flasks were chilled on ice for 10 min to remove the cells from the wall and harvested by centrifugation at 500× g for 5 min at 4°C. 5×10^5^ trophozoites ml^−1^ were transferred into induction medium (LG): TYI medium was prepared without glucose and diluted to 2.12 times with water and completed with 5% heat inactivated adult bovine serum, 2.6% vitamin mix and 125 μL/100 ml antibiotic. Cysts obtained in LG medium after three days were harvested and treated with 0.05% Sarkosyl (Sigma) to destroy the trophozoites. Typical spherical refractile cysts were observed by light microscopy and checked for staining with calcofluor and observed 80–90% encystation efficiency. For excystation the cysts were further washed with phosphate-buffered saline (PBS), and inoculated in normal TYI-S-33 medium.

### Transfection and selection of *E. histolytica* and *E. invadens* trophozoites

Transfection of *E. histolytica* was performed by electroporation as described previously [32]. Briefly, trophozoites in log phase were harvested and washed with PBS followed by incomplete cytomix buffer (10 mM K_2_HPO_4_/KH_2_PO_4_ (pH 7.6), 120 mM KCl, 0.15 mM CaCl_2_, 25 mM HEPES (pH 7.4), 2 mM EGTA, 5 mM MgCl_2_). The washed cells were then re-suspended in 0.8 ml of complete cytomix buffer (incomplete cytomix containing 4 mM adenosine triphosphate, 10 mM glutathione) containing 200 µg of plasmid DNA and subjected to two consecutive pulses of 3000 V/cm (1.2 kV/0.4 cm cuvette) at 25mF (Bio-Rad electroporator). The transfectants were initially allowed to grow without any selection. Drug selection was initiated after 2 days of transfection in the presence of 10 mg/ ml G-418. Transfection and selection of transfectants in *E. invadens* was done exactly as described earlier [33], [34].

### RNA purification and Real Time RT-PCR

*Entamoeba* cells were harvested and suspended immediately in TriZol reagent (Invitrogen) and RNA was isolated and treated with DNase I (Roche) according to manufacturer's protocol. RNA was reverse transcribed into cDNA using Superscript III reverse transcriptase (Invitrogen) and random hexamers used as a primer. Real-time RT-PCR was used to quantify HR and meiotic specific mRNA expression of *E. invadens* and *E. histolytica* during encystation and serum starvation respectively. This was carried out using an ABI real-time PCR system 7300 (Applied Biosystems) with Power SYBR Green PCR Master Mix. Primers were designed by ABI Primer Express 3.0 software. Each primer was analyzed against the *E. histolytica* and *E. invadens* database and any primer that had significant sequence similarity to multiple genes was rejected. Thus, both the forward and reverse primers were specific for one gene. Optimal annealing conditions were used to ensure specificity and any PCR primer pair that produced more than one melt peak was discarded.

The reaction mix contained 2 pmol gene-specific primers (Table S1in file S1), 10 μl SYBR green mix, and template cDNA in a volume of 20 μl in RNase-free water. The “comparative C_t_” method [35] was used to determine the relative quantities of the mRNA expression levels. 18S rRNA was used as the endogenous control which remained changed upon different treatments. In addition, EhTMKB1-9 (which is known to be down regulated during serum starvation) was used for comparison in real time PCR with *E. histolytica* [36]. A validation curve using serial dilutions of cDNA was used. After initial denaturation for 10 min at 95°C, the amplification cycle (repeated 40 times) was as follows: 15 s at 95°C 30 s at 55°C and 1 min at 60°C. The relative mRNA levels were expressed against those of trophozoites as 1.

### Southern and northern analysis

Southern and northern blotting was done according to established protocol [37]. RNA (30 µg) was size fractionated on a 1.2% denaturing agarose gel and subsequently blotted to a GeneScreen membrane (Perkin Elmer). Under stringent conditions, hybridization was done overnight at 65°C in hybridization buffer [1.0M NaCl, 1.0% Sodium Dodecyl Sulfate (SDS), and 10 µg/ml salmon sperm DNA] with the different gene specific DNA probes. The probes were prepared by PCR using the primers described in Table S1in file S1 and *E. histolytica and E. invadens* genomic DNA as template and labelled with α^32^P dATP (BRIT) by using NEblot KIT (NEB). The membrane was then washed at room temperature in 2x SSC, twice followed by 2x SSC and 1% SDS at 65°C for 10 min and with 0.1x SSC for 20 min at room temp, three times. The band intensities were quantitated by densitometry, and mRNA levels of each gene were expressed relative to 0 h (beginning of stress, or transfer to encystation medium). The data was average of three independent measurements.

### Inverted-repeat substrate construction

An inverted-repeat substrate was constructed as follows: The plasmid p*Ei*NEO/LUC [33] was used to provide a functional neomycin resistance cassette for selection. The sequence provided as inverted repeat was a 730 bp DNA sequence from *E. histolytica* intergenic region, with no similarity to *E. invadens* genome. The 730 bp DNA fragment was cloned in inverted orientation at the two ends of the LUC by using enzyme pairs *Xho*I/*Kpn*I and *Bam*HI/*Hind*III, in the *E. histolytica* vector p*Eh*NEO-LUC and *E. invadens* vector p*Ei*NEO-LUC. This construct was transfected in *E. histolytica* (and *E. invadens*) and stable cell lines were established. HR was measured by PCR-based determination of the amount of flipping. Different sets of primers were designed for measuring HR (P2+P3 in transfected cell lines and P1+P3 in inverted SINE sequences). PCR was performed with following program: 94°C/5 min, 25 cycles at 94°C/30 s, 51°C/30 s, 72°C/1 min and 72°C/7 min. Linearity of PCR was determined as shown with primer set P2+P4 (Fig. S1 in file S1).

## Results

### Meiotic and HR specific genes in *Entamoeba*

The presence of meiotic and HR specific genes in *Entamoeba* genome has been bioinformatically studied and reported [19], [20], [21], [38], [39]. However the data are mainly on *E. histolytica* genes and similar analysis with *E. invadens* is not available. We searched the Pathema (*Entamoeba*) database for these genes and found homologues of a number of putative meiotic genes (*DMC1, SPO11* and *MND1*) and HR specific genes (*MRE11, RAD21, RAD51* etc.) in *E. invadens*. The same genes are found in *E. histolytica* as well. Results of BLASTp analysis giving a list of all the identified genes in *E. invadens* and *E. histolytica* is given in Table 1. The known functions of each of these genes in yeast and human is given in Table S2 in file S1 [15], [16], [40]–[45]. Two copies of putative *SPO11* were found in *E. invadens* (*SPO11*a and *SPO11*b). The expression of these genes has been checked and reported in *E. histolytica* in response to DNA damage [21], [38]. We present below the expression analysis of some of these genes in *E. histolytica* in response to growth stress and in *E. invadens* during encystation.

**Table 1:** BLAST search of protein and nucleotide sequence databases at Pathema and AmoebaDB for meiotic- and HR-specific genes in *E.invadens* (Ei) and *E.histolytica* (Eh).

Gene Name	Pathema ID	Super family	Conserved Domain	Yeast			Human		
				E-Value	Maximum Identity (%)	Accession No.	E-Value	Maximum Identity (%)	Accession No.
***Eh*** **DMC1** ***Ei*** **DMC1**	EHI_050430EIN_ 249340	RecA_like NTPases	PTZ00035	1e-1221e -120	5252	NP011106. 1NP011106.1	3e-1369e- 137	6060	NP008999. 2NP008999.2
***Eh*** **SPO11** ***Ei*** **SPO1 1A** ***Ei*** **SPO11B**	EHI_125320EIN_ 220180EIN_137380	TP6A_N &TOPRIM	PRK04342COG1 697PLN00060	7e-073e- 043e-09	242926	EDV09027. 1EGA58462.1NP011841.1	4e-211e-232e- 15	272828	NP937998. 1NP937998.1NP036576.1
***Eh*** **MND1** ***Ei*** **MND1**	EHI_120310EIN_ 051380	COG5124	Mnd1	1e-112e- 08	2726	NP011332. 2NP011332.2	1e-533e- 47	4341	NP115493. 1NP115493.1
***Eh*** **MLH1** ***Ei*** **MLH1**	EHI_129950EIN_ 037260	HATPases_C &ToPoII_MutL_ trans	Mutl, MutL, mutL	2e-816e- 77	2927	ABC86942. 1AAA16835.1	1e-782e- 79	2642	NP001245200. 1NP001245200.1
***Eh*** **MRE11** ***Ei*** **MRE11**	EHI_125910EIN_ 156370	MPP	Metallophos	1e-399e- 43	2626	EGA60925. 1EDV11715.1	9e-581e- 56	3232	AAC78721. 1AAC78721.1
***Eh*** **RAD21** ***Ei*** **RAD21**	EHI_093880EIN_ 038720	Rad21_ Rec8_N	Putative conserved	2e-091e- 10	4431	GAA22237. 1CAA88356.1	6e-383e -41	5227	AAI57892. 1AAI57892.1
***Eh*** **MSH2** ***Ei*** **MSH2**	EHI_172750EIN_ 047820	MutS_III &ABC_ ATPases	MUTSd	3e-1212e -119	3938	AAA34802. 1CAY86200.1	3e-1289e- 123	3938	NP001245210. 1NP001245210.1

Values shown were obtained with BLASTp. *SPO11*, *DMC1* and *MND1* are meiotic specific genes.

### Expression of putative meiotic and HR specific genes in *E. histolytica* under normal and stressed conditions

**Figure 1:**
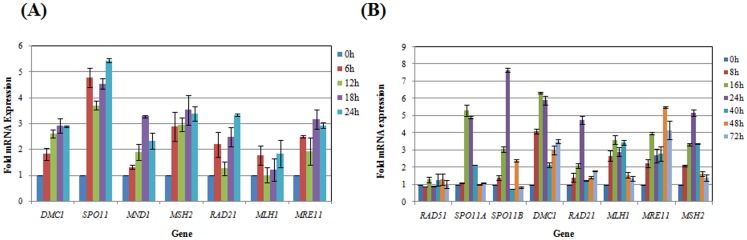
Expression of meiotic and HR specific genes during serum starvation in *E. histolytica* and encystation in *E. invadens*. (A) Real Time PCR analysis in *E. histolytica*. Total RNA was extracted at different time points (0, 6, 12, 18 and 24 h) during serum starvation using TRIZOL reagent (*Invitro*gen). cDNA was prepared by using Superscript III reverse transcriptase and qRT-PCR was performed with SYBR Green dye as a probe. 18S rDNA was used for normalization. The relative mRNA levels were expressed against those of trophozoites as 1. (B) Real Time PCR analysis in *E. invadens*. Total RNA was extracted at different time points (0, 8, 16, 24, 40, 48, and 72 h) during encystation and qRT-PCR was performed. mRNA levels of each gene were expressed relative to 0 h (beginning of encystation), shown in the bar graph. Size of all genes was checked by northern blotting (data not shown) by using appropriate probe, amplified by gene specific primers (Table S1in file S1). *SPO11*, *DMC1* and *MND1* are meiotic specific genes.

Cells subjected to a variety of stresses can suffer DNA damage, which is a trigger for HR. The expression of HR genes in response to induced DNA damage in *E. histolytica* has already been reported [21], [38], [39]. Serum starvation is known to induce a similar set of proteins as found during UV irradiation in HeLa cells [46]. Nutritional starvation in *Entamoeba* can be a trigger for differentiation of trophozoite to cyst [4], and it is therefore interesting to study cellular responses under these conditions. We have reported several distinct responses of *E. histolytica* cells to serum starvation, for example selective expression of a trans membrane kinase [36], and inhibition of pre rRNA processing with concomitant accumulation of a novel non coding circular RNA [47]. In this study we decided to check the effect of serum starvation on expression of HR genes in *E. histolytica*, by qRT-PCR analysis (Fig. 1A). For this we selected candidate genes from the major steps in the recombination pathway, viz., formation of DSBs (*SPO11*), sister chromatid pairing (*RAD21*), resection of ends following DSBs (*MRE11*), formation of nucleoprotein filament (*RAD51, DMC1*), recombinase enhancement by stabilizing the presynaptic filament (MND1), mismatch repair during strand invasion (*MSH2*), and resolution of HJs (*MLH1*).

RNA was extracted from normal *E. histolytica* trophozoites, and those subjected to serum starvation for different time periods (6-, 12-, 18- and 24-h of starvation) and gene expression was measured by quantitative RT-PCR and northern hybridization. 18S rRNA was used for data normalization. All the selected genes were expressed in normal trophozoites and were up-regulated during starvation (Fig. 1A). (*EhMLH1* could not be studied by northern hybridization as it was very poorly expressed, although the levels were detectable by qRT-PCR). Although each gene showed a distinct response to stress it was observed that, in general, all the genes were maximally expressed at 18 h and 24 h of serum starvation. After 24 h the cells began to undergo lysis. The general increase in mRNA abundance for all the tested genes was not due to a non-specific effect of starvation as we have earlier reported in our lab that mRNA levels of a trans-membrane kinase, EhTMKB1-9, go down in starved cells and simultaneously the mRNA levels of another member of the same family (EhTMKB1-18) begin to increase [36].

Expression levels of the tested genes went up between 1.2- to 5.5- fold during starvation. In some cases the expression level increased within 6 h of serum starvation, went down at 12 h and began to go up again. The relative expression of these genes was compared by northern blot analysis which showed that *EhSPO11, EhMND1, EhRAD21* and *EhMRE11* were expressed at low levels in normal proliferating cells whereas *EhDMC1* was expressed at moderate levels. The sizes of the bands in northern blots corresponded with the predicted gene sizes (data not shown).

### Expression pattern of putative meiotic and HR specific genes in *E. invadens* during encystation

Expression was checked by qRT-PCR analysis as described above. Cells were transferred to encystation medium, following which RNA was extracted after different time intervals (8-, 16-, 24-, 40-, 48-, and 72-h in encystation medium). As we have earlier shown, encystation under these conditions is complete by 72 h [8]. We routinely obtain 80–90% conversion of trophozoites to cysts as judged by resistance of cysts to sarkosyl, and staining of the chitin wall with calcofluor. Northern blot analysis showed that like *E. histolytica*, *EiSPO11*a and *EiSPO11*b were poorly expressed in normal *E. invadens* cells whereas *EiDMC1* was expressed at moderate levels (data not shown). The genes tested were all up-regulated during encystation, except *EiRAD51* whose levels did not change substantially. The expression went up substantially (2- to 7.8-fold) after 16–24 h of induction, after which it decreased and, in some cases, went up again (Fig. 1B). At 72 h when encystation is complete, the expression level of all genes dropped from the peak expression level of 16–24 h. The high level of expression of most of these genes at 16–24 h post induction indirectly shows that HR is likely to play an important role in the differentiation process.

The *DMC1* and *RAD51* genes share extensive regions of identity as they are derived from a common ancestor [48]. To obtain gene-specific PCR primers for these two genes (Table S1in file S1) primers were so designed as to have mismatches at the 3′-end with the other gene sequence. The amplicon obtained with each primer set was a single band of the size expected of the cognate gene.

### Demonstration of homologous recombination by using inverted repeat construct

**Figure 2:**
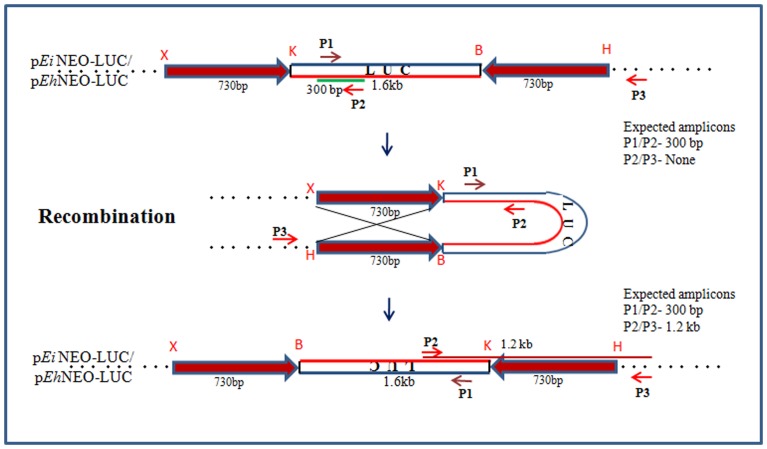
The inverted repeat substrate. A 730 bp segment was cloned in opposite orientation, flanking the 1.6*E. histolytica* vector p*Eh*NEO-LUC and *E. invadens* vector p*Ei*NEO-LUC. X: *Xho*I; K: *Kpn*I; B: *Bam*HI; H: *Hind*III. P1, P2, P3 are PCR primers (orientation indicated by arrows) used to measure inversion of the LUC sequence following recombination.

**Figure 3:**
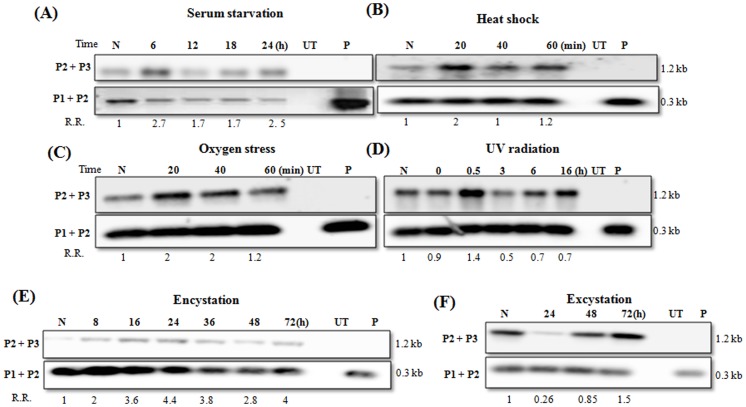
Demonstration of HR during growth stress in *E. histolytica* and encystation in *E. invadens*. Cells were transfected with the inverted repeat construct (Fig. 2). HR was demonstrated by measuring densitometrically the amplicon obtained with primer pair P2/P3 compared with primer pair P1/P2, by Southern analysis with LUC probe. Amplicon obtained by primer set P2/P3 was sequenced for confirmation of recombination (Fig. S2 in file S1). R.R.: Relative Recombination (ratio of amplicon intensity with P2/P3 versus P1/P2, with respect to normal trophozoites (N) as 1). Recombination was checked in different stress conditions i.e. (A) serum starvation; (B) heat stress; (C) oxygen stress and (D) UV irradiation with UV-C light (150 J/m^2^ ) for 8 sec followed by incubation in fresh medium for indicated time periods [21]; (E) *E. invadens* cells transferred to encystation medium and (F) excystation in fresh medium. UT: DNA from un-transfected cells; P: DNA of LUC plasmid. Full-length blots are presented in Fig. S4 in file S1.

From the data presented above it is evident that recombination-related genes are expressed in *Entamoeba* and are up-regulated during stress in *E. histolytica* and during encystation in *E. invadens*. However recombination itself has not been demonstrated in *Entamoeba* so far. This has been particularly difficult since genetic markers are not available for selection of recombinants, and attempts to knock out genes by homologous recombination have been unsuccessful. To circumvent the problem due to lack of genetic selection system in *Entamoeba* we decided to measure recombination by using repetitive sequences on a plasmid, as reported earlier for model systems [49], [50]. We introduced into *E. histolytica* and *E. invadens* cells (by stable transfection) a plasmid construct containing two identical inverted repeats (730 bp each) separated by a 1.6 kb fragment of luciferase (LUC) gene (Fig. 2 for details). Stable cell lines were obtained by G418 selection, and the plasmid was stably maintained in these cells. Recombination between inverted repeats on the plasmid would cause flipping of the intervening LUC sequence, which can be measured by PCR with appropriate primers. The primers P2/P3 are in the same direction in the original construct and do not give any amplicon with the parental plasmid (Fig. 2, and lane ‘P’ in Fig. 3). Recombination between the inverted repeats would place these primers in opposite orientation and result in a 1.2 kb amplicon from the recombined molecules. As an internal control the primer pair P1/P2 was used which would give a 300 bp amplicon both from the parental and recombined plasmids.

In mid log-phase cells, recombined molecules could be seen in all samples (Fig. 3 A-D, lanes N in each panel), as evidenced by the amplicon obtained with primer pair P2/P3, which gives no amplicon with the parent plasmid (lane P). Since the plasmid construct used for transfection gave absolutely no amplicon with primer set P2/P3, the amplicon obtained in the transfected cell line is not due to plasmid contamination in the DNA samples. The observed amplicon is also completely absent in the untransfected *Entamoeba* cells (Fig. 3, lane UT), showing that the primers used do not give spurious amplicons with *Entamoeba* DNA. The results obtained cannot be attributed to differential episome persistence or loss in different samples as the cells were stably transfected with the episome and were grown and maintained under continuous G418 selection. Moreover, the primer set P1/P2 provided the internal control for parental plasmid in each sample.

To conclusively show that the results are not due to PCR artifacts, and that the PCR amplicon obtained with primer pair P2/P3 indeed corresponded with the expected recombination event between the inverted repeats, we sequenced the amplicon (Fig. S2 in file S1). This clearly showed flipping of the sequence exactly at the expected recombination junction, providing direct evidence of recombination in *E. histolytica*, and showing that the plasmid construct was suitable for measuring recombination.

The ratio of amplicon intensity obtained from primer pairs P2/P3 and P1/P2 can be used to compare recombination efficiency in cells, before and after they are subjected to growth stress. This read out was used to measure the relative levels of recombined molecules in normal cells and those grown under different stress conditions (Fig. 3), as stress is thought to increase recombination [51], [52]. Comparing the ratio of PCR product (in the linear range of PCR, as shown in Fig. S1in file S1) obtained from primer pair P2/P3 versus P1/P2 in normal and serum-starved cells showed that the relative recombination efficiency increased 2.7-fold after 6 h of starvation and continued to be high till 24 h (Fig. 3A).

Cells exposed to other stresses (heat shock and oxygen stress) also showed increased HR within 20 min of stress exposure (Fig. 3B, C). Cells exposed to UV for 8 sec showed increased HR after 30 min of revival from UV treatment (Fig. 3D). Thus it is clear that HR takes place in *E. histolytica* and is activated during stress.

In *E. invadens*, HR was checked during encystation and excystation. The same primer sets were used as for *E. histolytica*. Genomic DNA was extracted at different time points during encystation and extent of recombination was checked by PCR. Relative recombination efficiency went up within 8h of transfer to encystation medium and remained high (3–4 fold) throughout encystation (Fig. 3E). *E. invadens* cysts were transferred to normal medium to induce excystation, and HR was examined. At 0 h of excystation i.e. in cysts, a high proportion of molecules were in the recombined configuration. Their number dropped at 24 h and again went up as growth proceeded (Fig. 3F), indicating that active recombination was taking place both during encystation and excystation.

### Demonstration of homologous recombination between endogenous copies of EhSINE1 repetitive sequences present as inverted repeats

**Figure 4:**
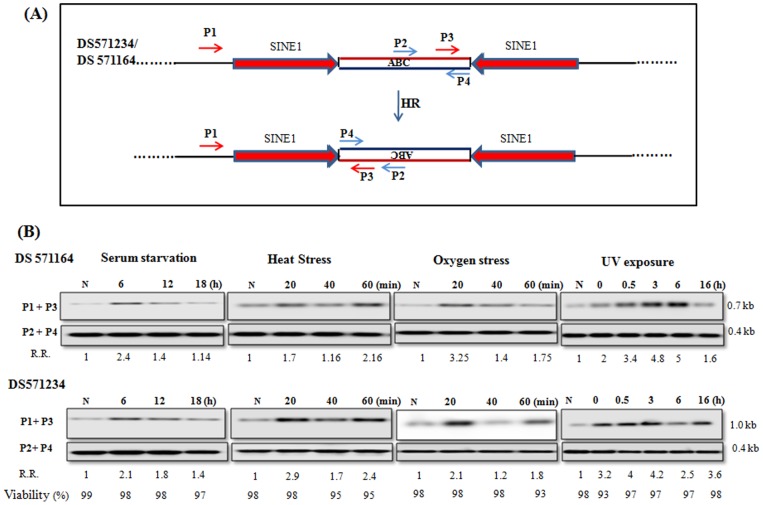
Demonstration of HR in the inverted copies of endogenous *Eh*SINE1. (A) Two pairs of *Eh*SINE1, separated by a 500bp spacer (scaffold DS571164) and 12 kb spacer (scaffold DS571234) which would get flipped after recombination. (B) HR was demonstrated with primer set P1/P3, which amplified a 0.7 kb product in DS571164 and 1.0 kb in DS571234 after HR event. Primer set P2/P4 gave 0.4 kb amplicon in both DS571164 and DS571234. Amplicon obtained by primer set P1/P3 was sequenced for confirmation of recombination (Fig. S3 in file S1). R.R.: Relative Recombination (with respect to unstressed cells). Viability of stressed cells was measured by trypan blue. Full-length blots are presented in Fig. S5 in file S1.

In the previous section we demonstrated HR between inverted repeats located on an extra-chromosomal plasmid. It is important to know whether such recombination also takes place in the context of the chromosome. For this purpose we decided to look at repetitive DNA copies, some of which may be located in an inverted orientation with respect to each other. Of the repetitive DNAs in *E. histolytica* genome the SINEs are very abundant [53]–[55]. The sequences of full length *Eh*SINE1 copies were extracted from the database [54], and closely related copies were selected. Of these, two pairs in which the *Eh*SINE1 copies were present in inverted orientation with respect to each other were selected. Pair I was in scaffold number DS571164 and the two copies had 85% identity, and pair II was in scaffold number DS571234 with the two copies being 100% identical. The SINE copies in pair I were separated by 500 bp and those in pair II by 12 kb. This intervening sequence should invert as a result of recombination. Primers P1/P3 were designed from the same strand such that they would amplify a 0.7 kb band (in pair I) and a 1.0 kb band (in pair II) if recombination had occurred, as shown schematically (Fig. 4A). The primer set P2/P4 was designed such that it would give a 0.4 kb amplicon in both SINE pairs I and II, from the molecules in the original configuration (as seen in the genome sequence), and used as an internal control. Recombinant products (from primer pair P1/P3) were confirmed by sequencing (Fig. S3 in file S1).

In exponentially growing cells most molecules were in the original configuration, as expected and genomic DNA gave an intense band with primer pair P2/P4 in each case (Fig. 4B). Amplicons of low intensity were obtained with primer pair P1/P3 also, showing that recombination of chromosomal copies does occur during the normal growth of *E. histolytica*. As observed with episomal construct in the previous section, the level of recombined molecules went up significantly during stress in this case also. In serum starved cells the intensity of P1/P3 amplicon went up after 6 h in both the SINE pairs, after which there was a drop. In heat stress and oxygen stress, the HR event appeared to be cyclic, being high at 20 and 60 min, and somewhat lower at 40 min. After brief exposure to UV, cells showed increased HR, which declined by 16 h (Fig. 4B). Since both loci showed the same behaviour it may be concluded that SINE loci, and possibly other genomic regions as well, undergo active HR, which is enhanced during growth stress.

## Discussion

HR is an accurate method for repair of DNA damage induced by environmental factors, or by intrinsic processes like the collapse of replication forks. It is essential for the maintenance of genomic integrity and is also required for chromosomal segregation during meiosis. For this reason HR is expected to be a highly conserved function in all organisms. However, in organisms like *E. histolytica* where genetic markers are not available and which do not show obvious sexual stages or DNA exchange, HR has not been directly demonstrated. Genome sequence analysis of *E. histolytica* strongly indicates the existence of HR in this organism. For one, homologues of a large number of genes belonging to HR machinery could be found in the *E. histolytica* database [20], [21]. Additionally, sequence analysis has provided some evidence of gene conversion in the gene encoding the Gal/GalNac lectin complex of *Entamoeba* [56]. Here we show that not only are the HR genes present in *Entamoeba*, they are also transcribed into mRNAs of the predicted size. For expression analysis we selected candidate genes from all major steps in the recombination pathway. qRT-PCR analysis showed that transcripts of all of these genes could be detected in normal cells and that these were up regulated during serum starvation in *E. histolytica* and under encystation conditions in *E. invadens*. Since DNA damage is a common consequence of growth stress, the observed up regulation of HR genes is physiologically significant.

The mere presence of homologous sequences of known HR genes from model organisms is insufficient to infer the presence of an active HR pathway in *E. histolytica* since these genes could well have roles in alternate pathways. On the other hand since a large number of HR-related genes have been maintained by selection in the *E. histolytica* genome it appears unlikely that all of them perform alternate functions in this organism. Thus the most straightforward interpretation is that these *E. histolytica* genes are involved in the conserved function of HR. A rigorous functional analysis of these gene products has not yet been done, although the DNA-binding and D-loop forming properties of the *E. histolytica* RAD51p have been documented [21].

We present here a PCR-based method using inverted repeats to measure recombination in *Entamoeba*. We believe our method gives a good read out of recombination as one can know the structure of the final recombination junctions by sequencing the amplicons. Once the stable transfectants containing the inverted-repeat plasmid construct are obtained the method is very convenient and rapid. This has provided the first direct demonstration that inverted repeat sequences, either located on a plasmid or chromosomally, are capable of recombining in *E. histolytica*. Additionally we show that recombination efficiency increases under stress, concomitant with the up regulation of HR genes. This further permits the conclusion that the HR-related genes in *E. histolytica* are likely to perform their known conserved functions. A general observation in the inverted repeat recombination reported here was that the population of recombined molecules increased rapidly in response to stress, followed sometimes by a decline and again an increase in their number. Both the appearance and disappearance of recombined molecules is indicative of recombination activity. Since the molecules in the original configuration are much more abundant, we did not expect to see a decline in the number of recombined molecules, once they were formed. We speculate that this could happen if there was a delay in the final resolution of Holliday junctions, and the resolution favoured the original, over the recombinant configuration. Alternatively, it is possible that the newly recombined molecules (due to some unknown features) may be the preferred targets of new rounds of recombination, thus resulting in reversal. In either case, the propensity to revert to the original configuration might explain the failure of many labs to obtain recombinant molecules during gene knock-out studies in *E. histolytica*. The immediate surge of recombination in *E. histolytica* cells subjected to a variety of stress conditions (serum starvation, heat and oxygen stress) can be exploited to assist in gene knock-out, which has so far not been successful in this organism. Our data suggests that better results may be obtained if the cells transfected with the suitable DNA construct for gene knock-out are exposed to a brief duration of stress.

The simple life cycle of *E. histolytica* and *E. invadens* consisting of the uninucleate trophozoite and the tetranucleate cyst shows no obvious indication of any sexual stage or exchange of genetic material. On the other hand, homologues of the major meiosis-specific genes (*SPO11, DMC1, RAD51, MRE11*) were not only found in both *E. histolytica* and *E. invadens*, we also report that these genes were transcriptionally active. The diplomonad *Giardia intestinalis* shares some basic features with *E. histolytica* in that both organisms inhabit the human intestine, both form dormant cysts with no obvious sexual stage, and both have metabolic similarities, as they grow optimally under the same culture conditions [57]. However the *Giardia* trophozoite consists of two nuclei, each of which is diploid, while the *E. histolytica* trophozoite consists of a single nucleus, which is thought to be tetraploid [27]. Studies with *Giardia* strongly indicate that DNA exchange occurs following nuclear fusion (karyogamy) during encystation [23]. Although chromosomal crossing over in a meiotic exchange to generate recombinant genotypes has not been demonstrated, several lines of evidence have been put forward to suggest that meiosis indeed takes place in *Giardia*. The presence of homologous gene sequences corresponding to the core meiotic machinery in *Giardia* and other protists (*Entamoeba*, *Plasmodium*, *Trypanosoma* and *Leishmania*) has been cited as evidence of an ancestral origin of meiosis and its central role in all extant eukaryotes [20]. Phylogenetic analyses of gene sequences from different isolates of *Giardia* showed topological differences in the trees obtained from different genes, which is indicative of recombination [58], [59]. However the data are not sufficient to differentiate between meiotic sex and parasex [60]. Similar studies need to be carried out with *Entamoeba* once sufficient sequence data from different isolates is available. The most likely stage at which genetic exchange may occur in *Entamoeba* could be the time at which the tetraploid uninucleate trophozoite gives rise to the tetranucleate cyst, a stage where our study shows enhanced recombination in *E. invadens*. Genetic tools need to be developed to further analyze HR using the *E. invadens* system. The recent demonstration of stable transfection in *E. invadens* is a step in this direction [33].

Another line of reasoning links the mode of reproduction of a species with its ability to sustain retrotransposons in its genome. Accordingly it is thought that organisms which reproduce solely by asexual means would eventually lose these elements from their genomes [61]. *E. histolytica* harbours a large number of non-LTR retrotransposons, which are distributed on all chromosomes, and account for ∼11% of the genome [53], [55], [62]. Active retrotransposition has recently been demonstrated in an *E. histolytica* cell line [63]. Although circumstantial, this further suggests that *Entamoeba* might engage in sexual exchange of DNA sequences at some stage in their life cycle. Further studies with *Entamoeba* and other early-branching protists are required to understand the origin of sex and meiosis and to discover the variety of mechanisms by which DNA exchange may occur in these organisms.

## Supporting Information

File S1 **Figure S1. Determination of optimum PCR cycles. Figure S2. Recombinant product with primer set P2+P3. Figure S3. Recombinant product with primer set P1+P3. Figure S4. Full blots of the data in** **figure 3** **. Figure S5. Full blots of the data in** **figure 4** **. Table S1. List of primers used for qRT-PCR. Table S2. Meiotic and homologous recombination specific genes.**
(DOCX)
