# Supplementary material for: Homologous Recombination Occurs in Entamoeba and Is Enhanced during Growth Stress and Stage Conversion
Source: PLoS One. 2013 Sep 30;8(9):e74465. doi: 10.1371/journal.pone.0074465 (PMC3787063; doi:10.1371/journal.pone.0074465)
Supplement: File S1 — Figure S1. Determination of optimum PCR cycles. Figure S2. Recombinant product with primer set P2+P3. Figure S3. Recombinant product with primer set P1+P3. Figure S4. Full blots of the data in figure 3 . Figure S5. Full blots of the data in figure 4 . Table S1. List of primers used for qRT-PCR. Table S2. Meiotic and homologous recombination specific genes. (DOCX) [file pone.0074465.s001.docx]

**Homologous recombination occurs in *Entamoeba* and is enhanced during growth stress and stage conversion**

Nishant Singh^1^, Alok Bhattacharya^2^ and Sudha Bhattacharya^1^*

1- School of Environmental Sciences, Jawaharlal Nehru University, New Delhi, India.

2- School of Life Sciences, Jawaharlal Nehru University, New Delhi, India.

Nishant Singh (nishant900@gmail.com)

Alok Bhattacharya (alok.bhattacharya@gmail.com)

Sudha Bhattacharya (sbjnu110@gmail.com)

*** Corresponding author**

Sudha Bhattacharya

E-mail: [sbjnu110@gmail.com](mailto:sbjnu110@gmail.com); [sb@mail.jnu.ac.in](mailto:sb@mail.jnu.ac.in)

Telephone: 91-11-26704308

**Figure S1. Determination of optimum PCR cycles.**

**Figure S2. Recombinant product with primer set P2+P3.**

**Figure S3a Recombinant product with primer set P1+P3.**

**Figure S3b Recombinant product with primer set P1+P3.**

**Figure S4 Full blots of the data in figure 3**.

**Figure S5 Full blots of the data in figure 4.**

**Table S1 List of primers used for qRT-PCR**.

| **Gene** | **Forward primers (5′ – 3′)** | **Reverse primers (5′ – 3′)** | **Amplicon size (bp)** | **Pathema ID** |
| --- | --- | --- | --- | --- |
| *Eh*dmc1*  *Eh*spo11*  *Eh*mnd1*  *Eh*mlh1  *Eh*mre11  *Eh*rad21  *Eh*msh2 | GTGGAGGGATTGAAACAATGAGTG  GGAGACGTCAAGTTACTATTTGGAG  CTGTCAAAAGAAGAGAAACGACAACG  GGACAAATAACAGAAAATCCAATTCCT C  TTGGTAAAGCAGAAGATATTTCAGAAATTG  GTCATTGATTGATACTACTATGGCAGG  GGAGGAAGTTACAACGGACAATGC | CCTTCAGTATCAATATAGCTACTTTTCCAT  AAATGGAGACGAAAGAATTGGTAGTTG  CTAACAATCCCTTTTTCTTTTGGTGC  TATGAATTGCATAGTCCCCAATTATCG  CTGAGATGATGCCCATAGTCTATTC  CATCTTCGTTAATCATTGGTAATGGTGC  GTCTATCAACGGTTGTTTAATCCATTC | 120  159  112  162  160  133  168 | EHI_050430  EHI_125320  EHI_120310  EHI_129950  EHI_125910  EHI_093880  EHI_172750 |
| *Ei*spo11A*  *Ei*spo11B*  *Ei*dmc1*  *Ei*mnd1*  *Ei*rad51  *Ei*rad21  *Ei*mre11  *Ei*mlh1  *Ei*msh2 | CTCATTCCAACAGTTCCCTTTC  CTAAAACACCACGAGGACTT AAAAGCTCGGGAAGATGATG  GCTGTGTCGAAAGAGGAGAAACTG CTGACGAGTTTTCTGTTGCTGTTGT  TCATCCACCCCGTAGACAAAGA GAAGAGGAAAACAGCGAAATAGC  AAAATTCCATAGATGCCAAAGC  TGATCAAAGTTTGGCGGTGTAT | CAAAAATATCGAGCCCGCATGG  CATCTCACGAACCTTTGACT  TGTTCGCCCTTCCCTTTTCT  CCTTTTTCTTTGCTGGCTACTACCTC  GATTCTCGACTCTCCTTTGCCTTT  CGCCCTTAAGTTCGGTTGTTC  ACTGCTTTTTCTAGACAATAATTGAC  CCTCCGATGTTTTTCGATGT  TCCTCTTCCCTTCAATCACTACTTG | 264  199  198  106  169  249  208  218  199 | EIN_220180  EIN_137380  EIN_249340  EIN_051380  EIN_136330  EIN_038720  EIN_156370  EIN_037260  EIN_047820 |

**Table S2 Meiotic and homologous recombination specific genes.**

| **Gene Function** |
| --- |
| ***SPO11**** Transesterase; creates DNA double strand breaks (DSBs) in meiosis I. ^[15^ **^]^**  ***DMC1**** Homolog of *RAD51*, form helical filaments on ss- and dsDNA and catalyses homologous DNA pairing and strand exchange ^[16, 40]^.  ***MND1**** Function after meiotic DSBs and required for stable heteroduplex DNA formation ^[41]^.  ***MRE11*** 3ʹ-5ʹdsDNAexonuclease and ssDNA endonuclease activity, trims back  broken DNA ends and hairpins ^[42,43]^.  ***MSH2*** Formation of hetero-dimer with *MSH3* or *MSH6* ^[44]^.  ***MSH6*** Forms a hetero-dimer with *MSH2*, binds base-base mismatches ^[44].^  ***MLH1*** Involved in mismatch repairing of di-nucleotide and tri-nucleotide sequences,  interact with *MSH2* and forms hetero dimer with *MLH2,MLH3* and *PMS1*^[44]^.  ***RAD21*** Involved in the holding of sister chromatids together during mitosis and meiosis ^[45]^.  ***RAD51*** Forms helical filaments on ss- and dsDNA and catalyzes homologous DNA pairing and strand exchange i.e. intra homologous recombination^16^. |
